# Blood Biomarkers for the Diagnosis of Peripheral Causes of Vestibular Syndrome: A Systematic Review and Meta-Analysis

**DOI:** 10.1097/MAO.0000000000004608

**Published:** 2025-09-12

**Authors:** Vincent W. Klokman, Merel J.J. Verhagen, Marieke S. Sanders, Korné Jellema, Dominique P.V. de Kleijn, Kim E. Jie

**Affiliations:** ∗ Department of Vascular Surgery, University Medical Center Utrecht, Utrecht, the Netherlands; † Department of Emergency Medicine, Jeroen Bosch Hospital, 's-Hertogenbosch, the Netherlands; ‡ Department of Neurology, Haaglanden Medical Center, The Hague, the Netherlands; § Department of Emergency Medicine, St. Antonius Hospital, Nieuwegein, the Netherlands

**Keywords:** Biomarker, Dizziness, Menière's disease, Meta-analysis, Peripheral, Systematic review, Vertigo, Vestibular neuritis

## Abstract

**Background:**

Peripheral vestibular syndromes (PVS) encompass disorders such as benign paroxysmal positional vertigo, vestibular neuritis, and Ménière's disease, presenting with vertiginous symptoms. Existing diagnostic approaches rely on clinical and vestibular function tests but have overlapping presentations and invasive investigations. Blood-based biomarkers may offer a minimally invasive diagnostic alternative.

**Objectives:**

This systematic review and meta-analysis aims to synthesize current evidence on blood-based biomarkers to identify PVS compared with controls.

**Methods:**

A literature search was conducted for studies until January 1, 2025, in PubMed, Ovid Medline, and EMBASE databases analyzing blood-based biomarkers for PVS versus controls. The QUADAS-2 assessed study quality and a meta-analysis was conducted on biomarkers reported by at least two studies. Pooled standardized mean differences (SMD) with 95% confidence intervals were calculated, and heterogeneity was assessed using the *I*^2^ statistic.

**Results:**

Thirty-four studies (2,857 PVS, 3,249 controls) met the inclusion criteria where 75 biomarkers were identified and 31 meta-analyzed. Significant differences between PVS and controls were identified for otolin-1 (SMD, 1.53; 95% CI, 0.95 to 2.12), 25-OH vitamin D (SMD, −0.47; 95% CI, −0.76 to −0.19), C-reactive protein (SMD, 0.86; 95% CI, 0.35 to 1.37), leukocyte counts (SMD, 0.45; 95% CI, 0.18 to 0.72), neutrophil counts (SMD, 0.85; 95% CI, 0.44 to 1.25), and the neutrophil-to-lymphocyte ratio (SMD, 0.80; 95% CI, 0.48 to 1.12).

**Conclusions:**

Inner ear–specific protein (otolin-1) and inflammatory markers (e.g., CRP, fibrinogen, leukocyte/neutrophil counts, and NLR) demonstrated potential diagnostic utility for PVS. Larger, prospective studies should confirm these findings, establish normative values, and explore combined biomarker panels to enhance diagnostic accuracy in PVS.

## INTRODUCTION

The peripheral vestibular syndrome (PVS) encompasses a range of disorders that present with vertigo, imbalance, and other sensorineural symptoms (1). These disorders include benign paroxysmal positional vertigo (BPPV), vestibular neuritis (VN), labyrinthitis, and Menière's disease (MD) (2). PVS can cause considerable morbidity, impacting patients' quality of life and daily functioning. Accurate and early diagnosis is essential to initiate timely management, reduce healthcare costs, and prevent complications such as falls, anxiety, and chronic dizziness (3).

Although the clinical history and bedside examination remain cornerstones for identifying peripheral vestibular disorders, substantial overlap in symptom presentation can complicate diagnosis. Despite advances in clinical evaluation—including vestibular function tests like the head impulse test, caloric stimulation, and vestibular-evoked myogenic potentials—definitive diagnosis can still be challenging (4).

Identifying reliable biomarkers, detectable in accessible bodily fluids or tissues, may increase diagnostic precision and allow for more targeted management (5). Blood-based biomarkers—protein, hormonal, or genomic/metabolomic biomarkers—could offer new avenues for early detection and objective confirmation of PVS. Heightened inflammatory processes, reflected by markers such as C-reactive protein (CRP), fibrinogen, or leukocyte-based parameters may characterize auto-immune or inflammatory disease (1). Specific inner ear proteins such as otolin-1 and other otolith-related proteins have, for instance, highlighted the potential to distinguish BPPV from healthy controls, as abnormal circulating levels of these proteins may reflect otoconial detachment or damage (6,7). Furthermore, alterations in circulating vitamin D levels, possibly contributing to disordered calcium metabolism in the vestibular apparatus, are emerging as another biomarker candidate in BPPV and other vestibular conditions (8).

Proteomic studies of inner ear diseases show promise in clarifying pathophysiological mechanisms, revealing novel biomarkers that could aid in differentiating peripheral vestibular lesions from central causes (9). Beyond BPPV, proteomic signatures may be integral to detecting and monitoring autoimmune or inflammatory processes that underlie labyrinthitis and vestibular neuritis (5). Identification of protein panels that are inner ear–specific (e.g., otolin-1, otoconin-90, prestin) raises the prospect of developing rapid, minimally invasive blood tests that support clinical diagnosis and treatment decisions (4). Evaluating the breadth of proposed inflammatory, metabolic, hormonal, and inner ear–specific biomarkers in a single review may help clarify which markers are most diagnostically promising and which require further inquiry.

This systematic review aims to synthesize current evidence on blood-based biomarkers used to identify peripheral vestibular syndromes compared with healthy controls. We will evaluate the methodological quality of existing studies and explore the diagnostic accuracy of candidate biomarkers. Our findings will serve as a resource for guiding evidence-based practice and informing further exploration of biomarker-based diagnostics in peripheral vestibular disorders.

## METHODS

### Study Design

This systematic review and meta-analysis included patients 18 years or older with symptoms of dizziness or vertigo due to peripheral vestibular disorders, assessing the diagnostic value of blood-based biomarkers. Comparisons were made against healthy controls, with outcomes including diagnostic accuracy metrics and standardized mean differences. The timing of biomarker collection is limited to acutely symptomatic patients in both outpatient and inpatient clinics. This study is registered in the PROSPERO International Prospective Register of Systematic Reviews (registration number CRD42024576350) and is reported in accordance with the PRISMA (Preferred Reporting Items for Systematic Reviews and Meta-Analyses) guidelines and checklist for diagnostic test accuracy (Supplementary File 1, http://links.lww.com/MAO/C153) (10,11).

### Search Strategy

A literature search was designed and conducted with the assistance of a medical librarian on January 1, 2025, using the electronic databases Ovid Medline, PubMed, SCOPUS, Cochrane, and EMBASE. Both MeSH and free-text keywords were used, covering all known diseases, common symptoms portrayal, and associated terms related to peripheral vestibular syndrome—such as “vestibular neuritis,” “vertigo,” “dizziness,” “vestibular diseases,” “biomarkers,” and “blood proteins.” The full search strategy is available in Supplementary file 2, http://links.lww.com/MAO/C154. The aim of the search was to identify studies investigating individuals experiencing dizziness, specifically those to distinguish peripheral vestibular disease from healthy controls. The term “PVS” was not used exclusively, given the adaptations in condition nomenclature (12). Additionally, references and citations from all included articles were reviewed to identify any other potentially eligible articles. Only articles in English or Dutch were included. No major deviations from the registered PROSPERO protocol were made.

### Eligibility Criteria

Eligible studies met the following criteria:1) cohort, case-control, retrospective-observational and/or diagnostic test study strategy; 2) individuals 18 years or older; 3) symptomatic with complaints due to a PVS condition (e.g., BPPV, MD, VN, or labyrinthitis) and compared with healthy controls; 4) determination of the biomarker through blood testing; 5) reported descriptive statistics as the mean and standard deviation (SD) or median, ranges, and/or interquartile range values (IQR); and 6) confirmed final diagnosis using standardized neurotologic evaluations and disease-specific diagnostic, as appropriate per disease as established by the Bárány Society (13–17).

Exclusion criteria included: 1) nonhuman studies; 2) case reports, editorials, or letters to the editor; 3) individuals with recent head trauma (<72 h); 4) studies assessing biomarker levels in samples other than blood; 5) studies not comparing PVS to a control group; and 6) failure to report biomarker concentration and ranges.

### Data Extraction

Two reviewers (V.K. and K.E.J.) independently screened all titles and abstracts for exclusion based on the predefined eligibility criteria. Full-text versions of the remaining citations were then retrieved electronically. From each included study, the reviewers independently extracted the following information: 1) bibliographic details; 2) clinical data, including study design, sample size, sample type, participant age, gender, and time of symptom onset; and 3) measurement data, such as biomarker values per group, statistical findings, and the use of a reference standard. Extracted data were compared, and any discrepancies were resolved through consensus among all authors.

### Risk-of-Bias Assessment

The quality and risk-of-bias assessment for all included studies were independently conducted by two authors (V.K. and K.J.) using the *Quality Assessment of Diagnostic Accuracy Studies-2* (QUADAS-2) tool and the *Cochrane Handbook for Systematic Reviews of Interventions* (18,19). Several a priori criteria were applied when evaluating each study's risk of bias and applicability across the domains of patient selection, criterion standard, index tests, reference standard, and flow and timing. Studies that used inappropriate exclusion criteria were rated as having a “high” risk of bias. Comparisons of PVS to central acute vestibular syndrome (AVS) were excluded to avoid bias due to high misdiagnosis rates seen in HINTS+ examination across physicians (20) and potential underreporting related to timing of MRI (21). Studies using diagnostic strategies inconsistent with the Bárány Society consensus documents (13–17) were also considered to have a high risk of (misclassification) bias and were excluded. Studies were included in the meta-analysis only if they were assessed as being of at least moderate quality and presenting a low risk of bias. Studies with multiple high risk of bias features were excluded. Risk-of-bias and applicability concerns are summarized in Table 1. Publication bias was intended to be investigated using funnel plot analysis (58).

**TABLE 1:**
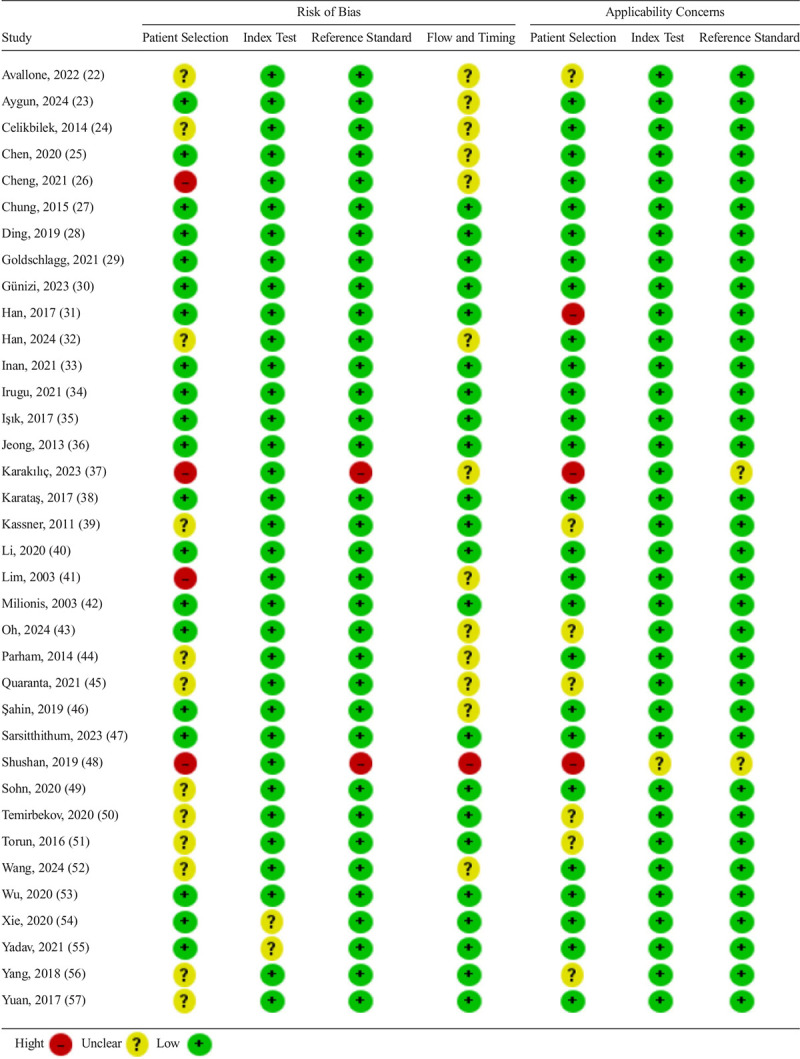
Risk-of-bias and applicability concerns summary table

### Data Analysis

A meta-analysis was performed for each biomarker if the mean and SD were reported in more than one study. Subanalyses were carried out for each of the included peripheral etiologies where possible (BPPV, MD, and VN). When only median, ranges, and/or IQR were provided, the mean and SD were estimated based on sample size, following the methods described by Wan et al. (59) and Luo et al. (60). Studies reporting values with significant skewness, as calculated according to Shi et al. (61), were excluded from meta-analysis. To account for variability in laboratory measurement methods, detection techniques, and units of measurement, the standardized mean difference (SMD) was used to express the difference in biomarker concentrations between controls and peripheral groups, relative to the variability within each study. Pooled SMDs and 95% confidence intervals (CIs) were calculated for the included studies. Heterogeneity was assessed using the *I*^2^ statistic and visually inspected via forest plots. If the *I*^2^ was below 50%, a fixed-effects model was applied; otherwise, a random-effects model was used. Statistical significance was defined as a *p* value <0.05. All meta-analyses were performed using Cochrane's Review Manager (Version 5.4.1) (62).

## RESULTS

### Search and Study Characteristics

**FIG. 1:**
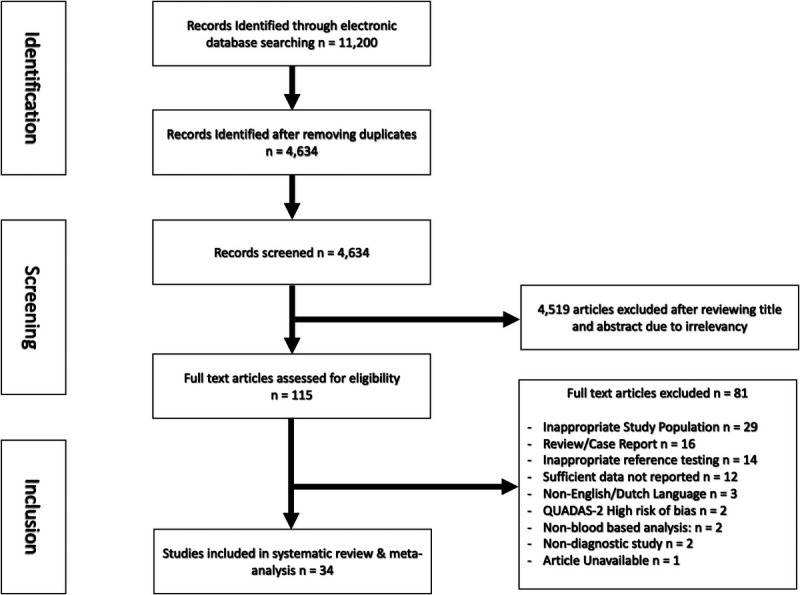
Preferred Reporting Items for Systematic Reviews and Meta-Analyses (PRISMA) flow diagram.

The literature search identified 11,200 articles. After removing duplicates across databases, 4,634 articles remained for title and abstract screening. Of these, 115 full-text articles were assessed for eligibility, resulting in 36 studies selected for inclusion. Following a risk of bias analysis (Table 1), two additional studies were excluded due to high risk of bias (37,48). We included 34 studies in the final review and meta-analysis (Table 2). Figure 1 presents the PRISMA flow diagram outlining the study selection process. A total of 2,856 patients with PVS were compared with 3,230 controls. The average age was 50.5 and 48.4 years for PVS and controls, respectively. The patient populations included 24 studies with BPPV, 4 studies with MD, 7 studies VN, and 1 study with VM subjects. Three studies investigated multiple PVS etiologies of PVS: two included both MD and VN (22,45), and one included BPPV and VN (49). A total of 69 blood biomarkers were analyzed, categorized into 13 hematologic markers, 46 clinical blood chemistry markers, and 10 organ-specific proteins (Supplementary File 3, Tables 1–3, http://links.lww.com/MAO/C155).

**TABLE 2:** Study and participant characteristics

Author, Year	Study Design	Target Condition	Sample Size PVS	Sample Size Control	Age PVS (yr)	Age Control (yr)	Gender PVS (M/F)	Gender Control (M/F)
Avallone, 2022	Prospective case-control	MD and VN	38	17	NR	NR	NR	NR
Aygun, 2024	Prospective case-control	BPPV	50	30	42.5	38.7	24/26	13/17
Celikbilek, 2014	Prospective case-control	BPPV	50	40	33.4	32.0	29/21	23/17
Chen, 2020	Prospective case-control	BPPV	90	90	55.22	55.17	28/62	28/62
Cheng, 2021	Prospective case-control	BPPV	320	320	68.2	68.9	144/176	153/167
Chung, 2015	Retrospective case-control	VN	70	70	49.57	49.57	31/39	31/39
Ding, 2019	Prospective case-control	BPPV	174	348	61	61	72/102	144/204
Goldschagg, 2021	Prospective case-control	BPPV	158	301	61	56	71/87	140/161
Günizi, 2023	Prospective case-control	BPPV	66	66	45.8	44.8	38/28	34/32
Han, 2017	Retrospective case-control	BPPV	85	80	63.5	63.9	0/85	0/80
Han, 2024	Prospective case-control	MD	33	176	60.8	61.1	13/20	69/107
Inan, 2021	Retrospective observational	BPPV	52	52	55.6	51.1	23/29	18/34
Irugu, 2021	Prospective case-control	BPPV	40	30	46.4	47	13/27	16/14
Isik, 2017	Prospective case-control	BPPV	64	63	56.2	57	17/47	18/45
Jeong, 2013	Prospective case-control	BPPV	100	192	61.8	60.3	37/63	92/100
Karataş, 2017	Prospective case-control	BPPV	78	78	51.4	48.9	29/49	33/45
Kassner, 2011	Prospective case-control	VN	12	12	57	49.6	NR	NR
Li, 2020	Prospective case-control	BPPV	204	120	38	38	103/101	60/60
Lim, 2003	Retrospective case-control	MD	26	31	50	35	11/15	NR
Milionis, 2003	Prospective case-control	VN	34	48	52.5	48	23/11	25/12
Oh, 2024	Retrospective case-control	VN	128	128	60.8	60.8	58/70	58/70
Parham, 2014	Prospective case-control	BPPV	14	10	NR	NR	0/14	0/10
Quaranta, 2021	Retrospective case-control	MD and VN	80	40	49	45	34/46	20/20
Sahin, 2019	Retrospective case-control	VN	104	138	44	43.5	51/53	63/75
Sarsitthithum, 2023	Prospective case-control	BPPV	69	68	61.4	60.0	10/59	17/51
Sohn, 2020	Prospective cohort	VN and BPPV	49	37	63.3	60.4	27/22	23/14
Temirbekov, 2020	Retrospective case-control	BPPV	142	135	44	42.2	48/94	34/71
Torun, 2016	Retrospective case-control	BPPV	35	32	53.2	47.7	22/13	19/13
Wang, 2024	Prospective case-control	VN	30	70	55	60	16/14	31/39
Wu, 2020	Prospective case-control	BPPV	78	121	62.7	61.4	52/26	61.4
Xie, 2020	Prospective case-control	BPPV	70	140	NR	NR	22/48	44/96
Yadav, 2021	Prospective case-control	BPPV	23	23	46.3	41.8	6/17	5/18
Yang, 2018	Prospective case-control	BPPV	50	52	NR	NR	0/50	0/52
Yuan, 2017	Prospective case-control	BPPV	240	72	62.4	63.5	106/134	39/33

BPPV indicates benign paroxysmal positional vertigo; MD, Meniere's disease; NR, not reported; VM, vestibular migraine; VN, vestibular neuritis.

### Single-Study Analyzed Biomarkers to Differentiate Between PVS and Controls

Biomarkers analyzed in single studies that were able to differentiate between PVS and controls include erythrocyte sedimentation rate (45), copper (30), disulphide (30), estradiol (56), interleukin-1β (25), lipoprotein A (42), native thiol (30), oxidized thiol (30), reduced thiol (30), thiol oxidation/reduction ratio (30), total thiol (30), soluble intercellular adhesion molecule-1 (25), soluble vascular adhesion protein-1 (25), superoxide dismutases (40), total bilirubin (54), and transthyretin (26).

Single-study analyzed biomarkers that were not able to differentiate PVS from controls include eosinophil counts (23), immature granulocyte percentage (23), systemic immune-inflammation index (23), antidiuretic hormone (41), apolipoprotein A–I (42), apolipoprotein B (42), brain-derived neurotrophic factor (49), creatine kinase-MB (51), follicle-stimulating hormone (56), glial fibrillary acidic protein (49), international normalized ratio (45), luteinizing hormone (56), neuron-specific enolase (49), parathyroid hormone (23), pre-albumin (57), progesterone (56), prostaglandin-E2 (25), S100 calcium-binding protein B (49), soluble CD40 (39), soluble CD40 ligand (39), total protein (51), tumor necrosis factor alpha (25), and zinc (30) (Supplementary Data 3, Tables 1 and 2, http://links.lww.com/MAO/C155).

### Meta-Analysis of Biomarkers to Differentiate PVS and Controls

**FIG. 2:**
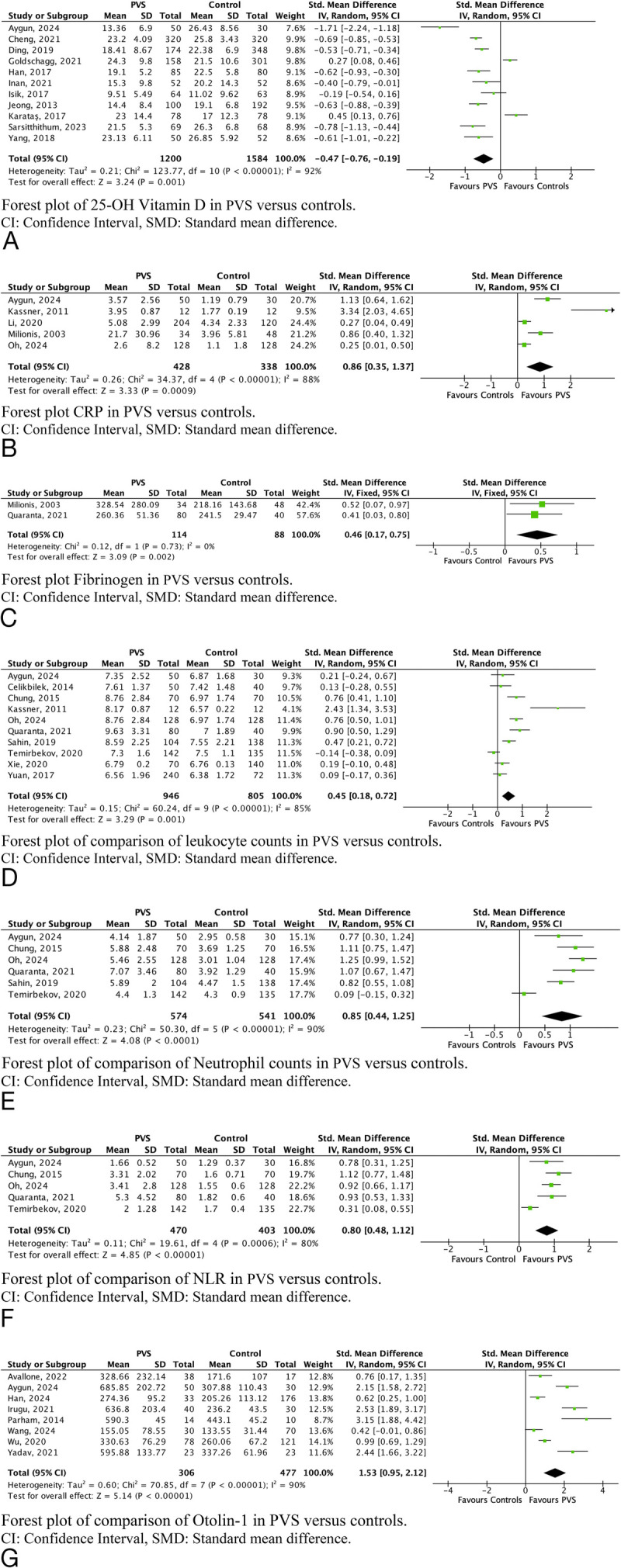
Forest plots for statistically significant markers between peripheral vestibular syndrome (PVS) and healthy control meta-analysis.

A total of 31 biomarkers were included in the meta-analysis, as presented in Table 3. Hematologic, clinical chemistry, and inner ear–specific markers that significantly differentiated PVS from controls included 25-OH vitamin D (SMD, −0.47; 95% CI, −0.76 to −0.19), C-reactive protein (CRP; SMD, 0.86; 95% CI, 0.35 to 1.37), fibrinogen (SMD, 0.46; 95% CI, 0.17 to 0.75), leukocyte counts (SMD, 0.45; 95% CI, 0.18 to 0.72), neutrophil counts (SMD, 0.85; 95% CI, 0.44 to 1.25), neutrophil-to-lymphocyte ratio (NLR; SMD 0.80; 95% CI, 0.48 to 1.12), and otolin-1 (SMD, 1.53; 95% CI, 0.95 to 2.12). These findings are visually presented as forest plots in Figure 2.

**TABLE 3:** Results from the meta-analysis of blood biomarkers to differentiate PVS from controls

Comparison Group	No. PVS Patients	No. Control Patients	No. Studies	SMD (95% CI)	*P* Value	*I*^2^ Value	Type of Model	Study Reference
25-OH vitamin D	1,200	1,584	11	−0.47 (−0.76 to −0.19)	0.001*	92%	Random effect	(23,26,28,29,31–33,35,36,38,47,56)
Albumin	715	604	5	−0.46 (−0.99 to 0.07)	0.09	94%	Random effect	(24,26,51,54,57)
ALT	440	500	3	−0.08 (−0.62 to 0.46)	0.77	91%	Random effect	(24,25,54)
AST	440	500	3	−0.36 (−1.18 to 0.46)	0.39	96%	Random effect	(24,25,54)
Blood urea nitrogen	275	104	2	−0.13 (−0.36 to 0.11)	0.29	0%	Fixed effect	(51,57)
CRP	428	338	5	0.86 (0.35 to 1.37)	0.001*	88%	Random effect	(23,39,40,42,43)
Calcium	166	145	3	0.05 (−0.42 to 0.51)	0.85	76%	Random effect	(23,33,35)
Creatinine	715	604	5	−0.90 (−2.04 to 0.24)	0.12	98%	Random effect	(24,26,51,54,57)
Fibrinogen	114	88	2	0.46 (0.17 to 0.75)	0.002*	0%	Fixed effect	(42,45)
Glucose	520	540	5	0.19 (−0.39 to 0.78)	0.75	98%	Random effect	(24,26,45,54)
HbA1c	560	392	2	−0.18 (−1.30 to 0.93)	0.75	98%	Random effect	(26,57)
HDL-C	761	664	7	−0.32 (−0.70 to 0.06)	0.1	89%	Random effect	(24,26,39,42,51,54,57)
Hematocrit	328	268	4	−0.18 (−0.54 to 0.18)	0.32	77%	Random effect	(23,27,43,45)
Hemoglobin	1,110	1,059	9	−0.08 (−0.16 to 0.01)	0.09	0%	Fixed effect	(23,24,26,27,43,46,53,54,57)
Homocysteine	560	392	2	−0.05 (−0.14 to 0.03)	0.13	0%	Fixed effect	(26,57)
LDL-C	761	664	7	0.40 (−0.29 to 1.09)	0.26	97%	Random effect	(35,37,38,41–43,54)
Leukocyte counts	946	805	10	0.45 (0.18 to 0.72)	0.001*	85%	Random effect	(23,24,27,39,43,45,46,50,54,57)
Lymphocyte counts	652	662	7	0.04 (−0.14 to 0.21)	0.69	69%	Random effect	(23,27,43,45,46,50,53)
Mean platelet volume	246	273	2	0.15 (−0.03 to 0.32)	0.1	70%	Random effect	(46,50)
Monocyte counts	120	100	2	0.51 (−1.04 to 2.06)	0.52	96%	Random effect	(23,27)
Neutrophil counts	574	541	6	0.85 (0.44 to 1.25)	<0.001*	79%	Random effect	(23,27,43,45,46,50)
Neutrophil to lymphocyte ratio	470	403	5	0.80 (0.48 to 1.12)	<0.001*	80%	Random effect	(23,27,43,45,50)
Otolin-1	306	477	8	1.53 (0.95 to 2.12)	<0.001*	90%	Random effect	(22,23,32,34,44,52,53,55)
Platelet counts	1,012	914	10	0.12 (−0.21 to 0.45)	0.49	90%	Random effect	(23,24,27,43,45,46,50,53,54,57)
Platelet-lymphocyte ratio	470	403	5	0.20 (−0.24 to 0.64)	0.38	90%	Random effect	(23,27,43,45,50)
Total cholesterol	841	704	8	0.10 (−0.11 to 0.32)	0.34	68%	Random effect	(24,26,39,42,45,51,57)
Triglycerides	521	592	6	0.39 (−0.35 to 1.13)	0.3	96%	Random effect	(24,26,39,42,51,57)
TSH	120	180	2	0.10 (−0.42 to 0.63)	0.7	77%	Random effect	(24,54)
Uric acid	715	604	5	0.01 (−0.40 to 0.41)	0.97	90%	Random effect	(24,26,51,54,57)

ALT, alanine transaminase; AST, aspartate transaminase; CI, confidence interval; CRP, C-reactive protein; HDL, high-density lipoprotein; LDL, low-density lipoprotein; PVS, peripheral vestibular syndrome; SMD, standard mean difference; TSH, thyroid-stimulating hormone.

Biomarkers that were not significantly different between PVS and controls include albumin, ALT, AST, BUN, calcium, creatinine, glucose, HbA1c, HDL-C, hematocrit, hemoglobin, homocysteine, LDL-C, lymphocyte counts, mean platelet volume, monocyte counts, platelet counts, platelet-to-lymphocyte ratio, total cholesterol, triglycerides, TSH, and uric acid (Supplementary data, Figs. 1A–V, http://links.lww.com/MAO/C155).

Otoconin-90 was analyzed in three studies. One study demonstrated significant differentiation between PVS and controls (*p* = 0.005) (32), whereas the other two did not (*p* = 0.892 (23) and *p* = 0.922 (52)). However, skewed data in all three studies precluded meta-analysis. Notably, 25-OH vitamin D, albumin, ALT, AST, BUN, calcium, creatinine, HbA1c, homocysteine, TSH, and uric acid were exclusively studied in BPPV patients.

### Assessment of Publication Bias

Funnel plot analysis was intended to assess publication bias. However, due to the limited number of studies per biomarker (none with >10 studies), meaningful assessment was not possible. Disease-stratified analyses were conducted to evaluate biomarker concentration differences across specific PVS subtypes (Supplementary Data 3, Figs. 2 and 3, http://links.lww.com/MAO/C155).

### Sensitivity Analyses Per Disease

**FIG. 3:**
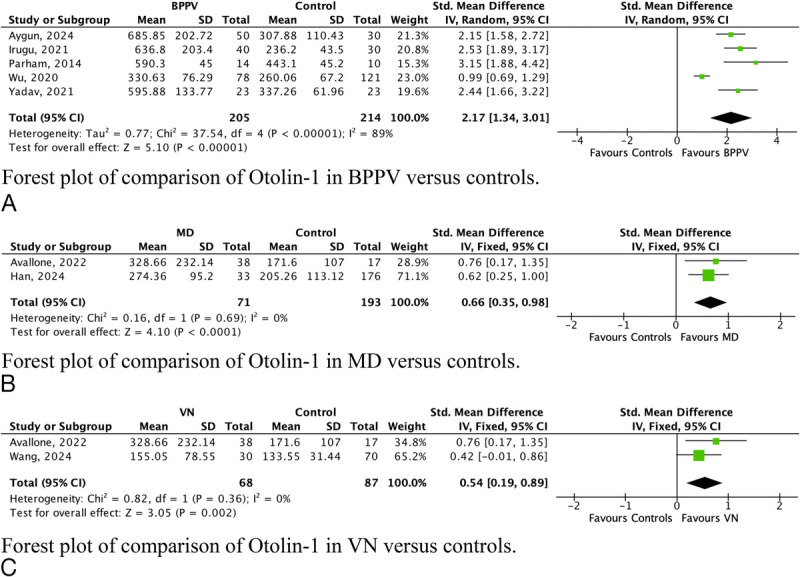
Forest plots of otolin-1 levels across benign paroxysmal positional vertigo. (BPPV), Menière's disease (MD), and vestibular neuritis (VN).

To evaluate whether disorder-specific heterogeneity could bias the pooled biomarker estimates, we repeated the analyses within separate subgroups for BPPV, MD, and VN. Otolin-1 remained significantly elevated across BPPV, MD, and VN groups (Fig. 3A–C), with the strongest association observed when pooled (SMD, 2.17; 95% CI, 1.34–3.01) compared with BPPV alone (SMD, 1.53; 95% CI, 0.95–2.12). In contrast, leukocyte and neutrophil counts were no longer significantly different in BPPV-only analyses, and the NLR showed a reduced effect size (SMD, 0.50; 95% CI, 0.05–0.95) compared with the overall analysis (SMD, 0.80; 95% CI, 0.48–1.12). Inflammatory and acute-phase markers including CRP, fibrinogen, leukocyte, and neutrophil counts remained elevated in VN patients (Supplementary File 3, Fig. 3, http://links.lww.com/MAO/C155). Subgroup analysis identified no additional biomarkers with a significant difference between peripheral etiologies.

## DISCUSSION

In our systematic review and meta-analysis, we evaluated 75 blood-based biomarkers across 35 studies. We performed a meta-analysis of 31 biomarkers, which provided evidence that inflammatory and hematologic parameters (e.g., CRP, fibrinogen, leukocyte counts, neutrophil counts, and NLR as well as inner ear–specific proteins [e.g., otolin-1]) significantly differed between PVS and control groups. Although reviews have analyzed the feasibility of specific blood-based biomarkers in certain subpopulations such as BPPV (7,8,63), sensorineural hearing loss (1), or central versus peripheral vertigo (64), to our knowledge, this is the first review and meta-analysis of blood-based biomarkers utilizinusing a syndrome approach to identify PVS compared with healthy controls.

### Blood-Based Biomarkers for PVS Etiology and Future Considerations

#### Inner Ear Markers

Our findings highlight otolin-1 as a promising biomarker capable of distinguishing peripheral vestibular syndromes (PVS) from healthy controls. Several included studies demonstrated significantly elevated otolin-1 levels in PVS, corroborating previous evidence that this protein reflects inner ear pathology (22). Data suggest that otolin-1 measurement may be confounded by age-related otoconia degeneration; one study showed that otolin-1 can rise with advancing age, emphasizing the need for robust normative data (65).

Although otoconin-90 was also investigated in multiple studies, meta-analysis was not possible due to skewed or insufficient data reporting. Preliminary results indicated conflicting outcomes regarding its discriminatory power, highlighting the necessity of larger, methodologically consistent investigations to fully establish its diagnostic utility. On the other hand, inner ear–specific markers such as otolin-1 continue to show broad potential, but results across specific diseases vary. For example, one study found no significant difference in otolin-1 or prestin levels between MD and VM, although comparisons to healthy controls were lacking (66).

Proteomic studies of inner ear diseases show promise in clarifying pathophysiological mechanisms, revealing novel biomarkers that could aid in differentiating peripheral vestibular lesions from central causes (9). The use of inner ear–specific biomarkers in diagnosing peripheral conditions in acute vestibular syndrome (AVS) could enhance diagnostic accuracy for peripheral etiologies while also potentially aiding in the exclusion of central causes, thereby improving clinical decision making.

#### Inflammatory and Hematologic Markers

Inflammatory and hematologic parameters featured prominently as potential diagnostic indicators. Several markers—CRP, fibrinogen, leukocyte counts, neutrophil counts, and the NLR—were significantly elevated in patients with PVS compared with controls. These results underscore the notion that peripheral vestibular disorders often involve a measurable systemic immune or inflammatory response. In particular, NLR not only appears to differentiate PVS from healthy controls but may also reflect the severity of vertigo, as elevated NLR correlated with higher Dizziness Handicap Inventory scores (67). This finding implies that ongoing inflammatory activity may interact with disease burden, underscoring the importance of further investigations to clarify how NLR behaves across varying clinical stages or subtypes of PVS. Recent immunologic research has proposed additional avenues for biomarker development. One study reported an association between VN and anti-ganglioside antibodies (68), although the lack of a control group limits definitive conclusions. These findings nonetheless reinforce the possibility that autoimmune mechanisms could be relevant in certain vestibular pathologies, providing an impetus for more detailed immunologic profiling in future studies.

Overall, combining inflammatory markers (e.g., CRP, NLR) with inner ear–specific biomarkers (e.g., otolin-1) may enhance diagnostic accuracy. Future prospective investigations, ideally with standardized sampling intervals and disease stage classifications (acute versus interictal), should evaluate these markers simultaneously. Larger-scale studies that define normative ranges for age and disease severity, along with the influence of comorbidities, will be crucial. Such efforts may pave the way for a multimarker diagnostic panel that accelerates timely, precise identification of peripheral vestibular disorders. Proteomic studies of inner ear diseases hold promise for clarifying underlying pathophysiological mechanisms and identifying novel biomarkers that may help distinguish peripheral vestibular lesions from central etiologies.

### Limitations

Despite measures taken to avoid bias and inclusion of studies of at least moderate quality, several limitations are nonetheless present. First, methodological variability across the included studies—ranging from differences in patient selection to the lack of standardized biomarker measurement intervals—may have biased pooled effect sizes and introduced potential biomarker time-concentration variance. While we excluded high-risk-of-bias articles, the overall risk could still be underestimated due to incomplete reporting in certain studies. Furthermore, the mean and SDs of several biomarkers were estimated from the reported median and IQRs and thus are not the exact values. We used the SMD to pool studies that reported biomarkers in different units or with different assays. Because the SMD expresses effects in SD units, a statistically significant SMD does not automatically indicate a clinically meaningful change, and its magnitude cannot be interpreted on the same scale as pooled sensitivities or specificities. Additionally, full access to raw data is necessary to calculate pooled sensitivities and specificities, with missing data potentially introducing additional bias. Several of the markers in the meta-analysis showed high levels of heterogeneity, though a random-effect model was used whenever this was the case. Disease staging and severity were inconsistently reported, precluding a more in-depth analysis of biomarker fluctuations over time. Finally, funnel plot assessment for publication bias was not feasible in most biomarkers because of the limited number of studies.

## CONCLUSIONS

In summary, this systematic review and meta-analysis underscores the potential diagnostic roles of both inflammatory (CRP, fibrinogen, leukocyte counts, neutrophil counts, and NLR) and inner ear–specific (otolin-1) biomarkers in PVS. These markers could refine the clinical workup for patients presenting with acute vertigo or dizziness, complementing conventional bedside neurotologic and audiovestibular evaluations. We recommend that future investigations adopt a prospective, multicenter diagnostic-accuracy design with standardized biomarker assays, clearly defined vestibular end-points, and direct head-to-head comparisons with current clinical algorithms to determine the true incremental value of each biomarker.
